# Assessment of Oxidative Damage to Proteins and DNA in Urine of Newborn Infants by a Validated UPLC-MS/MS Approach

**DOI:** 10.1371/journal.pone.0093703

**Published:** 2014-04-02

**Authors:** Julia Kuligowski, Isabel Torres-Cuevas, Guillermo Quintás, Denise Rook, Johannes B. van Goudoever, Elena Cubells, Miguel Asensi, Isabel Lliso, Antonio Nuñez, Máximo Vento, Javier Escobar

**Affiliations:** 1 Neonatal Research Group, Health Research Institute Hospital La Fe, Valencia, Spain; 2 Leitat Technological Center, BioInVitro Division, Valencia, Spain; 3 Department of Pediatrics, Division of Neonatology, Erasmus Medical Centre-Sophia Children's Hospital, Rotterdam, The Netherlands; 4 Department of Pediatrics, Free University Medical Centre Amsterdam, Amsterdam, The Netherlands; 5 Department of Pediatrics, Emma Children's Hospital, Academic Medical Center, Amsterdam, The Netherlands; 6 Department of Physiology, University of Valencia, Burjassot, Valencia, Spain; 7 Division of Neonatology, University & Polytechnic Hospital La Fe, Valencia, Spain; Instituto de Investigación Sanitaria INCLIVA, Spain

## Abstract

The assessment of oxidative stress is highly relevant in clinical Perinatology as it is associated to adverse outcomes in newborn infants. This study summarizes results from the validation of an Ultra Performance Liquid Chromatography–tandem Mass Spectrometry (UPLC-MS/MS) method for the simultaneous quantification of the urinary concentrations of a set of endogenous biomarkers, capable to provide a valid snapshot of the oxidative stress status applicable in human clinical trials, especially in the field of Perinatology. The set of analytes included are phenylalanine (Phe), para-tyrosine (p-Tyr), ortho-tyrosine (o-Tyr), meta-tyrosine (m-Tyr), 3-NO_2_-tyrosine (3NO_2_-Tyr), 3-Cl-tyrosine (3Cl-Tyr), 2′-deoxyguanosine (2dG) and 8-hydroxy-2′-deoxyguanosine (8OHdG). Following the FDA-based guidelines, appropriate levels of accuracy and precision, as well as adequate levels of sensitivity with limits of detection (LODs) in the low nanomolar (nmol/L) range were confirmed after method validation. The validity of the proposed UPLC-MS/MS method was assessed by analysing urine samples from a clinical trial in extremely low birth weight (ELBW) infants randomized to be resuscitated with two different initial inspiratory fractions of oxygen.

## Introduction

Reactive Oxygen Species (ROS) are free radicals and other chemically reactive molecules formed by partial reduction of oxygen involved in many physiological processes by modulating the functionality of redox sensitive proteins in a reversible manner [1]. ROS can be generated either by endogenous sources such as the mitochondrial respiratory chain and xanthine and NADPH oxidases or by exogenous sources such as UV-light, radiation or xenobiotics [2]. A large number of different ROS are known in biological systems, the most relevant being superoxide anion (O2^.−^), hydrogen peroxide (H_2_O_2_), hydroxyl radical (HO), and peroxynitrite (ONOO^−^). Excessive amounts of ROS may lead to oxidative stress defined as a disruption of thiol redox circuits leading to aberrant cell signaling and dysfunctional redox control that occurs under normal conditions [3]. ROS mediate oxidative damage to nucleic acids, lipids, carbohydrates, and proteins. Under normal circumstances, oxidative damage is counteracted by enzymatic repair mechanisms. However, under pathologic conditions ROS may induce irreversible oxidation of proteins, nucleic acids, lipids and carbohydrates leading to further clinical deterioration [4].

Non-enzymatic phenylalanine (Phe) hydroxylation mediated by HO. leads to the formation of ortho-tyrosine (o-Tyr) [5], [6] and meta-tyrosine (m-Tyr) isomers [6]. Both, 3-nitrotyrosine (3NO_2_-Tyr) and 3-chlorotyrosine (3Cl-Tyr) are oxidized para-tyrosine (p-Tyr) derivatives formed by peroxynitrite (ONOO^−^) or hypochlorous acid (HClO), respectively [5]. Altogether, these molecules are considered reliable biomarkers of oxidative damage to proteins [5]–[10]. Besides, 3NO_2_-Tyr and 3Cl-Tyr are also considered biomarkers of nitrosative stress, and inflammation and myeloperoxidase (MPO) activity, respectively [7]–[9]. DNA can also be a target for the action of superoxide anions which may cause oxidation of both deoxyribose phosphate backbone and the nucleo-bases. 8-hydroxy-2′-deoxyguanosine (8OHdG) (also known as 8-oxo-2′-deoxyguanosine (8-oxodG)), produced by the oxidation of the nucleotide 2′-deoxyguanosine (2dG), is commonly used as biomarker of oxidative damage to DNA [10]–[13].

Nevertheless, aerobic organisms have developed antioxidant defenses capable of effectively scavenge ROS [14]. In the mammalian newborn placental transfer and fetal synthesis of antioxidants occurs late in gestation in preparation for transition into the extra uterine milieu [2], [15], [16], [17]. Especially two circumstances among others such as prematurity and birth asphyxia aggravate oxidative stress due to oxygen overload [16]–[18]. Hence, it has been shown that resuscitation of term and preterm infants with high oxygen concentrations causes oxidative stress [2]. In addition, preterm infants are endowed with an immature antioxidant defense system which is only partially responsive to antenatal steroids. Therefore, preterm infants are extremely sensitive to hyperoxia and prone to develop oxygen free radical derived conditions that have short- and long-term consequences in terms of mortality and morbidity [19], [20]. Assessment with reliable oxidative stress biomarkers in preterm infants is therefore becoming a fist line field of research in Perinatology. In this context the use of urinary oxidative biomarkers avoiding invasive sampling techniques renders especially suitable for preterm infants [9], [10], [21]–[23]. ILCOR 2010 recommendations for restricting oxygen use in preterm infants were based on experimental and clinical studies that used urinary biomarkers to assess oxidative stress [24]–[26]. In addition, Escobar *et al.* [27] recently showed a correlation of o-Tyr, 8OHdG and m-Tyr with erythropoietin levels in amniotic fluid of pregnant women with type 1 or insulin-dependent gestational diabetes. Thus, amniotic fluid oxidative biomarkers may be used in the future to assess the severity of fetal hypoxia.

A number of procedures for the quantification of Phe, p-Tyr, halogenated 3Cl-Tyr and 3NO_2_-Tyr metabolites in urine, mainly based on gas chromatography (GC) and liquid chromatography (LC) with mass spectrometric (MS) detection can be found in literature [7]–[9]. LC-MS [28]–[30], LC-electrochemical detection (LC-ECD) [31] as well as GC-MS [32] have been proposed for the measurement of 8OHdG in urine. However, analytical methods targeting newborn infants should be adapted to their very specific characteristics (e.g. troublesome sample collection, specific concentration ranges).

The aim of this study was to develop and validate a rapid, non-invasive and straightforward UPLC–MS/MS method for the quantification of eight selected urinary biomarkers (o-Tyr, m-Tyr, p-Tyr, 3NO_2_-Tyr, 3Cl-Tyr, Phe, 2dG and 8OHdG) capable of providing a snapshot of the oxidative stress status of newborns. In addition, the analysis of a large number of samples from a recent clinical trial under the acronym REOX [EUDRACT 2088-005047-42] allowed us to determine the range of urinary concentrations of these biomarkers in extremely low gestational age newborns. The REOX trial is a multi-center double-blinded randomized clinical trial for studying the effects of resuscitation in ELBW infants initiated with low oxygen concentration (30%) versus high oxygen (60%) concentration. The objectives of this clinical trial were to increase the survival rate and reduce the occurrence of significant morbidities typically observed in premature infants, such as e.g. severe retinopathy of prematurity and bronchopulmonary dysplasia. The degree of oxidative stress experienced during fetal to neonatal transition, reoxygenation and stabilization has an ample range. However, the selected set of biomarkers has been capable of detecting significant changes even under a broad range of pro-oxidant conditions. Moreover, in order to have a normal pattern of urinary biomarker elimination the proposed analytes were also quantified using the same UPLC-MS/MS method in a control group of healthy term newborn infants born by vaginal delivery and not needing intervention in the delivery room.

## Materials and Methods

### Ethics Statement

The study protocol was approved by the local IRB (Institutional Review Board) of Hospital Universitario y Politécnico La Fe (Valencia; Spain) and also the CEICs (Comités de Ética e Investigación) of both University Maternity Hospital Casa de Salud (Valencia; Spain) and Hospital General de Sagunto General (Valencia; Spain). Parents/guardians of all participating neonates signed written informed consent.

### Solvents and standards

Acetonitrile (LC-MS grade), methanol (LC-MS grade) and formic acid (analytical grade) were purchased from Sigma Aldrich Quimica SA (Madrid, Spain). Water was Milli-Q grade (>18.2 MΩ) from a Millipore purification system. Standards of o-Tyr, m-Tyr, Phe, 3NO_2_-Tyr, 3Cl-Tyr, p-Tyr, 8OHdG and 2dG (>96% w/w purity) were obtained from Sigma-Aldrich (St. Louis, MO, USA). Deuterated phenylalanine (Phe-D_5_) was purchased from CDN Isotopes (Pointe-Claire, Canada).

### Infant's urine sample collection and pretreatment

Urine samples were obtained from very low birth weight infants in a randomized and blinded clinical trial carried out in the Division of Neonatology of Hospital La Fe (Valencia, Spain) which aimed to compare the safety and efficacy of initiating resuscitation with lower (30%) versus higher (60%) initial inspiratory fractions of O_2_ in the delivery room (REOX, 2012-2013, EUDRACT 2088-005047-42). Enrolled patients were newly born preterm infants with a gestational age of < 32 weeks. Twenty-two healthy newborn term babies with a gestational age of > 37 weeks and born by spontaneous vaginal delivery were also included in the study for assessing the suitability of the method in a control group. Urine samples were obtained by using sterile Hollister collection bags. Samples were centrifuged, aliquoted and stored at −80°C until processed. Samples were allowed to thaw on ice to minimize biological degradation of analytes during the sample preparation process. Then, they were homogenized by shaking on a Vortex mixer during 20s and centrifuged at 4°C and 7500 rpm (Biocen 22 R, Orto Alresa, Madrid, Spain) for 10 min to remove large particles. 100 μL aliquot of supernatants were acidified with 10 μL of H_2_O (0.5% v/v formic acid) and 5 μL of 10 μmol/L Phe-D_5_ internal standard solution were added. For UPLC-MS/MS analysis, samples were loaded on 96-well plates and analyzed randomly without having access neither to the physiological conditions nor to the resuscitation procedure followed for each patient.

### Preparation of stock, working and standard solutions

Individual stock solutions of 8OHdG (1 mM), Phe-D_5_ (1 mM), m-Tyr (2 mM), o-Tyr (2 mM), 3NO_2_-Tyr (2 mM), 3Cl-Tyr (2 mM), 2dG (2 mM), p-Tyr (10 mM), and Phe (10 mM) were prepared by accurately weighing the standards and dissolving in H_2_O (0.1% v/v HCOOH). Once prepared, each stock solution was aliquoted and stored at −20°C in capped amber vials. Stock solutions went through a single freeze and thaw cycle. Working solutions obtained by dilution of the stock solutions in H_2_O (0.1% v/v HCOOH) were used for preparing the standards and for spiking of samples. Working solutions were kept at −20°C in capped amber vials and went through a single freeze and thaw cycle. Standard solutions were prepared by serial dilution of the working solutions in the concentration intervals summarized in Table 1. Concentration ranges were selected from concentrations found in urine samples during a pre-validation study (results not shown).

**Table 1:** Figures of merit obtained from the calibration curves used for quantitation.

Analyte	Calibration range	Retention Time (min)^a^	Concentration range (LLOQ – ULOQ)	R^2,^ b	LOD (nM)	LOQ (nM)
p-Tyr	0.4 – 200 μM	1.452±0.003	0.44 – 220 μM	0.996	39.0	129.9
m-Tyr	16 – 250 nM	1.889±0.012	17 – 275 nM	0.996	1.2	3.9
o-Tyr	16 – 1000 nM	2.733±0.009	17 – 1100 nM	0.998	2.8	9.5
Phe	0.2 – 200 μM	3.100±0.003	0.22 – 220 μM	0.9991	14.4	48.0
3NO_2_-Tyr	16 – 1000 nM	3.415±0.004	17 – 1100 nM	0.995	4.2	14.0
3Cl-Tyr	7 – 2000 nM	3.211±0.008	8 – 2200 nM	0.997	0.9	3.0
2dG	7 – 1000 nM	2.51±0.02	8 – 1100 nM	0.996	0.7	2.5
8OHdG	2 – 250 nM	3.263±0.010	2.2 – 275 nM	0.993	0.2	0.6

a : mean values ± standard deviation;

b : Regression coefficient for the linear regression curves calculated using (Peak Area Metabolite)/(Peak Area of the Internal standard).as response, and ((Metabolite)/(Internal standard)) as independent variable. Internal standard: Phe-D_5_

#### Preparation of spiked samples

A pooled urine sample was prepared by mixing 250 μL of a total of 14 urine samples collected from preterm newborns. After homogenization, the pooled sample was aliquoted and kept at −80°C until its use. Each aliquot went through a single freeze and thaw cycle. For each validation batch aliquots of both, pooled urine sample and test samples were thawed on ice, homogenized by shaking on a Vortex mixer during 20s and centrifuged at 4°C and 7500 rpm for 10 min. 20 μL of three working solutions of increasing concentrations were added to 200 μL aliquots of supernatant for the preparation of the low, medium and high level spiked samples. Due to the lack of a blank urine sample for the recovery assessment, the mean values of basal levels of the analytes in the pooled sample were used for the recovery calculations (see Table 2).

**Table 2:** Concentrations of the analytes in the pooled urine sample.

Analyte	Intra-Day (mean±s)	Inter-Day (mean±s)
p-Tyr	89.6±0.4 μM	90 ±2 μM
m-Tyr	16.6±0.7 nM	16.5±0.4 nM
o-Tyr	49±1 nM	54±9 nM
Phe	37.2±0.5 μM	37±2 μM
3NO_2_-Tyr	<LOD	<LOD
3Cl-Tyr	<LOD	<LOD
2-dG	76.2±0.7 nM	75±4 nM
8OHdG	5.4±0.2 nM	6±1 nM

Note: Intra-day concentrations are the mean (±standard deviation) of three independent replicate analysis of the pooled sample carried out on the first validation day. Inter-day mean and standard deviation values were calculated using the concentrations found in three independent batches of three independent replicate analysis of the pooled sample

### UPLC-MS/MS analysis

UPLC-MS/MS analysis was carried out on an Acquity – Xevo TQ system (Waters, Milford, MA, USA) using positive electrospray ionization (ESI) and the following conditions: capillary 0.50 kV, cone 21.00 V, extractor 3.00 V, source temperature 120 °C, desolvation temperature 400 °C, nitrogen cone and desolvation gas flows were 50 and 750 L h^−1^, respectively. Multiple reaction monitoring (MRM) was carried out using the instrumental parameters summarized in Table 3. MS parameters (i.e. dwell time, cone voltage and collision energy) were optimized for each analyte by analyzing individual standard solutions at a concentration of 500 μmol/L. Dwell time was set to 5 ms to ensure a minimum of 10 data points per peak. Separation conditions were selected to achieve appropriate chromatographic retention and resolution by using an Acquity UPLC BEH C18 reversed phase column (2.1×50 mm, 1.7 μm) (Waters, Milford, MA, USA) and a CH_3_OH (0.05% v/v HCOOH):H_2_O (0.05% v/v HCOOH) binary gradient. Flow rate, column temperature and injection volume were set at 0.4 mL min^−1^, 30°C and 5 μL, respectively. The gradient employed was as follows: from 0 to 1 min 0% v/v CH_3_OH (0.05% v/v HCOOH) (i.e. channel B), from 1 to 2.5 min from 0 to 15% v/v B and from 2.5 to 4.5 from 15 to 99% v/v B. Conditions were maintained for 0.25 min followed by returning to initial conditions between 4.5 and 5.1, which were held for 0.9 min for system re-equilibration. During batch analysis, samples were kept at 4°C in the autosampler. An injection volume of 5 μL was used.

**Table 3:** MS/MS acquisition parameters.

			m/z daughter ion			
Analyte	m/z parent ion	Cone (V)	Quantification	CE (eV)	Confirmation	CE (eV)
p-Tyr	182.1	30	136.1	15	119.2	15
m-Tyr	182.1	30	136.1	15	-	-
o-Tyr	182.1	30	136.1	15	-	-
Phe	166.1	25	120.1	15	103.1	15
3NO_2_-Tyr	227.1	25	181.2	15	-	-
3Cl-Tyr	216.1	25	170.1	15	157.1	15
2dG	268.0	15	152.0	10	-	-
8OHdG	284.1	20	168.1	15	-	-
Phe-D_5_	171.1	35	125.0	10	-	-

For Phe, 3Cl-Tyr and p-Tyr two multiple reaction monitoring (MRM) transitions, one for quantification and the other one for confirmation, were monitored (see Table 3). Confirmation of the identity of these analytes in urine samples was based on the following criteria: i) both UPLC-MS/MS peaks must have the same retention time; ii) the relative abundance of the MRM signals at the peak apex must be within ±25% of the signal observed in a standard with a similar concentration; and iii) the MRM transitions must have signal-to-noise ratios (SNR) higher than 3. For all other analytes identification was based on the retention time due to the low intensity of additional MS/MS fragments.

Data was acquired and processed using MassLynx 4.1 and QuanLynx 4.1 (Waters, Milford, MA, USA), respectively. Linear response curves were calculated employing Phe-D_5_ as internal standard.

### Method Validation

The validation of the method was based on the FDA guidelines for bioanalytical method validation [33] and it was carried out using a standard operation procedure (SOP) drafted in advance. The SOP described all the relevant parameters of the method from sample collection to analysis. The quality parameters (i.e. figures of merit) incorporated in the validation included linearity range, precision, accuracy, selectivity, limit of detection (LOD) and quantification (LOQ), dilution integrity as well as sample and standards stability.

Responses were fit to linear regression with 1/x weighting. Acceptance criteria for each regression fit were as follows: i) calibration curves should include at least 6 points excluding the blank, ii) % relative standard deviations (%RSD) of standard replicates (n = 3) at three concentrations (i.e. low, mid and high) should be below 15%, iii) back-calculated accuracy had to be within ±20% of the nominal value and iv) coefficients of determination (R^2^) should be higher than 0.99. Nonetheless, as the use of R^2^ as linearity test might be misleading, a visual analysis of the calibration residuals was carried out for evidence of non- linear behavior [34]. Precision was determined at three levels (i.e. low, mid and high) in both, standards and spiked samples by calculating the RSD % of replicates (n = 3) within one validation batch (intra-day). Accuracy was established by comparing nominal and observed concentrations in spiked pooled urine samples at the concentrations summarized in Table 4. The non-spiked pooled urine sample employed was analyzed by triplicate on each validation day and results obtained were used for the calculation of the recovery values. Selectivity was evaluated by analyzing solvent blanks as well as non-spiked and spiked pooled urine samples. The LOD and LOQ values were estimated as the concentrations providing a signal to noise ratio of three and ten. The accuracy and precision in samples originally above the upper limit of quantification (ULOQ) was assessed by analyzing spiked samples at 2.5 times the concentration of the ULOQ following the above mentioned procedure and after a 1∶5 dilution in H_2_O (0.1% HCOOH).

**Table 4:** Back calculated accuracy and precision levels of calibration standards and spiked samples during the validation.

Analyte	Calibration standards Recovery ±s (concentration)			Spiked samples Recovery ±s (spiked concentration)^a^		
	Low	Medium	High	Low	Medium	High
p-Tyr	122±4 (0.8 μM)	86±4 (12.5 μM)	95±1 (150 μM)	90±5 (110 μM)	111±3 (140 μM)	123±2 (170 μM)
m-Tyr	98±6 (31 nM)	100±5 (125 nM)	98.9±0.6 (188 nM)	86±1.6 (80 nM)	90.0±0.5 (130 nM)	97±3 (200 nM)
o-Tyr	103±9 (31 nM)	95±3 (250 nM)	99±3 (750 nM)	96±4 (200 nM)	100.2±0.5 (475 nM)	100.7±0.8 (725 nM)
Phe	109±4 (0.4 μM)	100±2 (12.5 μM)	99±1 (150 μM)	98±2 (60 μM)	100.6±0.2 (115 μM)	100.8±0.7 (170 μM)
3NO_2_-Tyr	104±9 (31 nM)	93±7 (250 nM)	98±3 (750 nM)	77±4 (180 nM)	93±5 (455 nM)	91.6±0.3 (725 nM)
3Cl-Tyr	107±6 (16 nM)	89±3 (250 nM)	99±3 (1500 nM)	99.8±1.5 (180 nM)	100.3±0.8 (910 nM)	101.0±0.9 (1600 nM)
2-dG	109±8 (16 nM)	88±5 (125 nM) 9.70	98±2 (750 nM)	99±1 (250 nM)	103±1 (530 nM)	101±2 (725 nM)
8OHdG	106±10 (4 nM)	88±8 (31 nM)	98±3 (188 nM)	67±3 (80 nM)	81±2 (120 nM)	81±6 (190 nM)

a : concentrations in the spiked samples were calculated after subtracting the mean levels (n = 3) measured in the non-spiked pooled urine samples during each validation batch.

Analyte stability after three freeze-thaw cycles and the short-term stability (i.e. 24h at 4°C) were assessed on a standard solution and 5 non-spiked samples. Instrument performance was ensured by analyzing a mid-calibration standard by triplicate at the start of each validation batch. Carry-over effect between injections was evaluated by analyzing a blank solution directly after the injection of the highest concentrated standard.

### Analytical quality assurance

An initial system suitability test was carried out at the beginning of each batch to ensure the following items: i) the backpressure ripple at the beginning of the chromatographic run was <3%; ii) selectivity was ensured at the lower limit of quantification (LLOQ) for both blank sample (urine sample processed without internal standard) and a solvent (i.e. H_2_O (0.05% v/v HCOOH)) injection; iii) the instrument provided adequate sensitivity and precision levels. Hence, a standard solution at the lower limit of quantification (LLOQ) was analyzed by triplicate to ensure that the %RSD of the peak area values was ≤25% and the signal to noise ratios were ≥15 for each analyte; (iv) retention times did not vary more than ±0.2 min between consecutive batches.

Blank samples and solvent blanks were analyzed at the beginning of the sample batch measurement, after a high concentration standard and during the sample batch measurement to check for potential sources of contamination (e.g. column, mobile phase additives) and/or cross contamination. A spiked urine sample was intercalated in the sample batch measurement and used as quality control (QC) sample to detect deficiencies in accuracy and precision levels prior to the release of results. Accordingly, at least 75% of the values found for the QC standards should be within ±25% of their respective nominal values to accept the batch.

## Results

### Method validation

The validation of the UPLC-MS/MS method for the quantitative analysis of a subset of oxidative stress urinary biomarkers in newborns was carried out based on FDA guidelines [33]. Accordingly, the validation parameters included linearity range, precision, accuracy, selectivity, LOD, LOQ, dilution integrity as well as sample and standards stability. Additionally carry-over, retention time stability and instrument precision, were determined to assess instrument performance.

**Figure 1:**
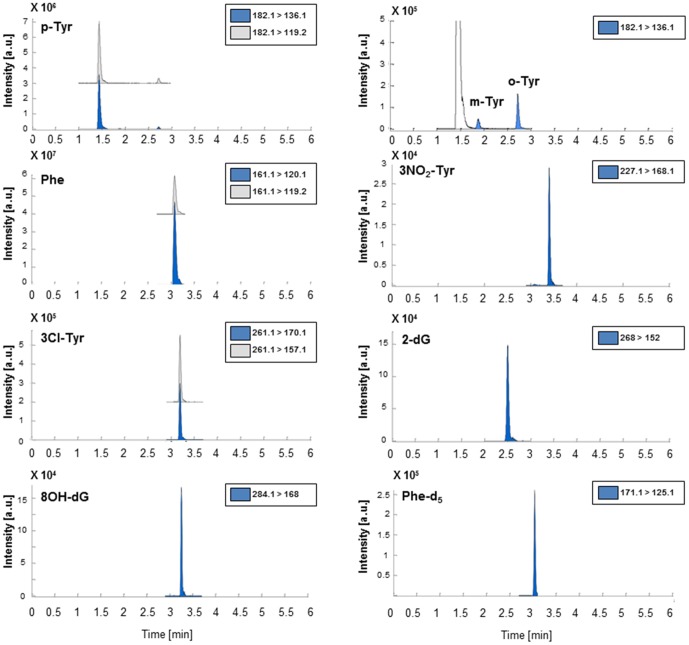
Typical chromatograms of the selected biomarkers extracted from the analysis of spiked urine sample. Note: Spiking concentrations were 73 nmol/L for 8OhdG and m-Tyr, 182 nmol/L for 2-dG, o-Tyr, 3NO_2_-Tyr and 3Cl-Tyr and 23 μM for p-Tyr and Phe.

Figure 1 shows representative UPLC-MS/MS chromatograms used for quantification extracted from the analysis of a spiked pooled urine sample. The main figures of merit obtained from the calibration curves including the concentration ranges of standards are summarized in Table 1. Retention time was found to be highly stable during the whole validation study with an intra-day standard deviation of the retention time of <0.02 min. Besides, no interfering peaks were observed from the comparison of UPLC-MS/MS chromatograms of blanks, non-spiked and spiked urine samples. System suitability was ensured by analyzing a middle calibration standard by triplicate at the start of each validation batch providing in all cases responses with RSD% values below 15%. The carry-over effect of the auto sampler was evaluated at the start of each validation batch by analyzing a blank injection directly after the injection of the highest concentrated standard. Results obtained showed that, for all MRMs included in the UPLC-MS/MS method, responses were lower than 5% of the lower LOQ (LLOQ) standard response, thus confirming the lack of a carry-over effect. As shown in Table 1, linear curves were calculated including a number of standards ranging between 6 and 12 for the different analytes. Both, the obtained coefficients of determination (R^2^≥0.993) and the homoscedasticity of the residuals (results not shown) were consistent with pre-established criteria, thus indicating appropriate linear responses in the selected concentration intervals. LODs for the measurement of protein and DNA oxidation products were ranging between 0.2 and 4.2 nmol/L. Precision and back-calculated accuracies obtained for standards at three concentration levels are summarized in Table 4. Recoveries were within ±20% deviation from the nominal value thus complying with the acceptability requirements. Precision values were also within the acceptability range indicating a high repeatability of the method. Table 4 summarizes the accuracy and precision values obtained from the analysis of spiked urine samples at three concentration levels (low, medium and high). As shown in this table, accuracy and precision levels of o-Tyr, m-Tyr, p-Tyr, Phe, 3NO_2_-Tyr, 3Cl-Tyr and 2dG fulfilled the pre-defined acceptance criteria. On the other hand, recoveries for 8OHdG at the low level were below the acceptability threshold (≥80%).

A dilution integrity test was carried out to assess the accuracy of the method in those samples in which the analytes were above the upper limit of quantification and needed to be diluted. Accordingly, three replicated spiked pooled urine samples were prepared to reach concentration levels 2.5 times above the upper LOQ (ULOQ). Then, the samples were diluted 5 times and analyzed. The back calculated concentrations of the oxidative stress biomarkers were within 75–117% of their nominal concentration with %RSD values ranging between 0.8 and 4%.

On storage at ‘auto sampler conditions’ (i.e. 24 h at 4°C), the concentration of the analytes from a mid-concentration standard was in the 97±2% range with respect to a freshly prepared standard. Again, no statistically significant differences were observed in the recovered concentrations of the analytes in both, standards and spiked samples after three freeze and thaw cycles, with deviations in their concentrations below 4%.

### Analysis of urine samples of extremely low birth-weight infants

Samples collected in a clinical assay aiming to reduce the oxygen load for preterm babies during postnatal resuscitation [REOX, 2012–2013, EUDRACT 2088-005047-42] were analyzed following the proposed procedure with a double objective: i) the assessment of the performance of the method for the analysis of real samples, and ii) the identification of reference concentration ranges for the selected metabolites in urine samples from preterm newborns.

**Figure 2:**
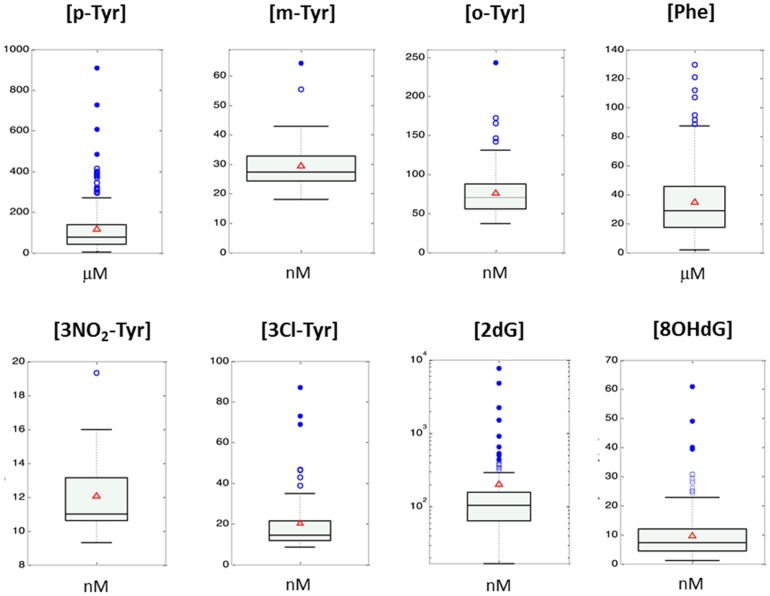
Non-zero concentrations found from the analysis of 222 urine samples of extremely low birth-weight infants included in the double-blinded randomized clinical study REOX (REOX 2012-2013, EUDRACT 2088-005047-42). The percentages of concentrations<LOD in the sample set were: 15% for o-Tyr, 79% for m-Tyr, 0% for p-Tyr and Phe, 93% for NO_2_-Tyr, 68% for 3Cl-Tyr, 0.4% for 8OH-dG and 0.4% for 2dG. Boxes indicate the 1^st^ and the 3^rd^ quartiles, the median is shown as a black line, whiskers mark the 9^th^ and 91^st^ percentiles, red triangles represent mean concentrations and blue circles are outliers.

**Figure 3:**
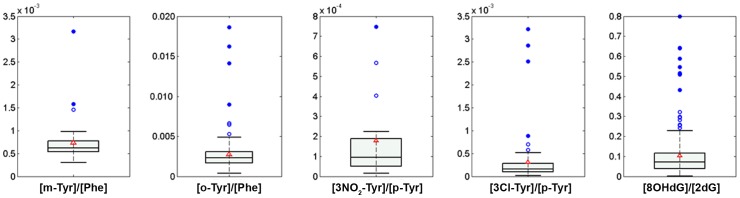
Boxplots of the ratios of o-Tyr/Phe, m-Tyr/Phe, 3NO_2_-Tyr/p-Tyr, 3Cl-Tyr/p-Tyr and 8OHdG/2dG for the set of non-zero urine samples included in the REOX clinical study. Boxes indicate the 1^st^ and the 3^rd^ quartiles, the median is shown as a black line, whiskers mark the 9^th^ and 91^st^ percentiles, red triangles represent mean concentrations and blue circles are outliers.

Figure 2 displays box plots of non-zero concentrations found from the analysis of 222 urine samples of extremely low birth-weight infants included in the REOX clinical study [EUDRACT 2088-005047-42]. The percentages of concentrations<LOD in the sample set were: 0.4% for 8OH-dG; 0.4% for 2dG; 93% for NO2-Tyr; 68% for 3Cl-Tyr; 15% for o-Tyr; 79% for m-Tyr, and 0% for p-Tyr and Phe. All detected concentrations were within the calibration ranges, not requiring reanalysis after sample dilution. Due to existing difficulties regarding normalization of the absolute concentrations of the analytes among different individuals, the ratio of the oxidation products and their precursors are typically reported, facilitating the biological interpretation of the figures. Figure 3 shows boxplots of ratios of o-Tyr/Phe, m-Tyr/Phe, 3Cl-Tyr/p-Tyr, 3NO_2_-Tyr/p-Tyr and 8OHdG/2dG found for the set of urine samples included in the clinical study.

### Analysis of urine samples of healthy term newborns

Urine samples of healthy breastfed term newborn babies (gestational age 37 to 40 weeks' gestation) born by vaginal delivery without instrumentation after uncomplicated gestation and with normal Apgar score at 1 and 5 min after birth at the University and Politechnic Hospital La Fe (Valencia; Spain), University maternity Hospital Casa de Salud (Valencia; Spain) and Hospital General de Sagunto (Valencia; Spain) were collected and analyzed following the proposed procedure to establish reference basal urinary ranges for the selected biomarkers. Table 5 lays out the range, median and mean values for the concentration of the selected biomarkers in the control group as well as the corresponding o-Tyr/Phe, 3NO_2_-Tyr/p-Tyr and 8OHdG/2dG ratios. The urinary concentrations of m-Tyr in the control group were lower than the LOD and therefore it was not possible to calculate the 3Cl-Tyr/p-Tyr ratio for healthy term newborns.

**Table 5:** Results control group (n = 6) consisting of term newborn infants born by vaginal delivery and lacking perinatal complications.

	Range	Median	Mean ± s	% Zero Samples	p-Value
p-Tyr (μM)	9.8-464.6	71.1	105 ± 111.9	0	0.2
m-Tyr (nM)	-	-	-	100	<0.001
o-Tyr (nM)	12.4-92	29.1	40 ± 24.7	0	<0.001
Phe (μM)	9.2–68.6	32.6	35.1 ± 19.3	0	0.4
3NO_2_Tyr (nM)	1.7–9.9	3.3	4.2 ± 2.5	82	0.4
3Cl-Tyr (nM)	5.1–14.5	10.3	10.1 ± 4.6	91	<0.001
2dG (nM)	18–422.8	84.4	133.1 ± 123.3	0	0.06
8OHdG (nM)	1.7–37.9	8.5	10.7 ± 8.1	0	0.14
m-Tyr/Phe	-	-	-	100	<0.001
o-Tyr/Phe	1.9*10^-4^ – 2*10^−3^	1.3*10^−3^	1.3*10^−3^ ± 5*10^−4^	0	<0.001
3NO_2_Tyr/p-Tyr	4*10^−6^ – 9*10^−5^	2*10^−5^	3*10^−5^ ± 3*10^−5^	82	0.003
3Cl-Tyr/p-Tyr	9*10^−5^–1.3*10^−4^	1*10^−4^	1.1*10^−4^ ± 1.3*10^−5^	91	<0.001
8OHdG/2dG	0.014–0.5	0.1	0.12 ± 0.08	0	0.15

Note: p-values correspond to two-groups (i.e. control vs extremely low birth-weight infants included in the study) comparisons using Student's t-test for independent data and assuming unequal variances. m-Tyr has not been detected in any sample.

## Discussion

Newborn infants, especially preterm infants, are prone to oxidative stress related diseases, which unequivocally influence their mortality, morbidity and long-term outcomes [20], [35]. Urine analysis has been considered to be a suitable alternative to e.g. invasive blood-based procedures. Besides, protein content in urine is lower and therefore sample preparation for LC-MS analysis is simpler than that needed for other bio-fluids (e.g. serum or plasma) [36].

The UPLC-MS/MS method for the quantification of eight selected urinary biomarkers (o-Tyr, m-Tyr, p-Tyr, 3NO_2_-Tyr, 3Cl-Tyr, Phe, 2dG and 8OHdG) was validated following FDA guidelines for bioanalytical method validation [33]. However, the FDA guideline aims at the analysis of drugs and their metabolites in a biological matrix and it cannot be fully applied to the analysis of endogenous metabolites. Whereas the guideline recommends the preparation of the calibration curve in the same biological matrix as the samples by spiking the matrix with known concentrations of the analytes, in this study the standards were prepared in H_2_O (0.05% HCOOH) due to the lack of suitable blank matrices. However, the assessment of the main figures of merit of the method including linearity range, precision, accuracy, selectivity, LOD and LOQ, dilution integrity as well as sample and standards stability, provided satisfactory results.

Representative chromatograms depicted in Figure 1 from the analysis of a spiked pooled urine sample, showed adequate selectivity and resolution among the analytes. Linear response was assessed in the selected concentration intervals with LODs in the nmol/L range ensuring a broad applicability of the method and a high repeatability of the retention times facilitating reliable analyte identification (see Table 1). Calculated concentrations of protein and DNA oxidation products from the analysis of spiked urine sample at three levels showed adequate precision and back-calculated accuracies. Recovery values were within ±20% deviation from the nominal value with the exception of 8OHdG at the low level (67±3%). Besides, dilution integrity test provided back accuracies in the 75–117% range with %RSD values ranging between 0.8 and 4%. These results ensured the applicability of the method in samples in which concentrations of the target analytes are up to 2.5 times above the upper LOQ (ULOQ), thus increasing its applicability. The stability of the analytes at ‘auto sampler conditions’ (i.e. 24 h at 4°C) was confirmed as the observed variation in the response of the analytes was within the range attributed to the instrumental imprecision. Besides, analyte concentrations were found to be stable in both, standards and spiked samples after three freeze and thaw cycles, with deviations in the concentrations below 4% facilitating the sample collection and storage before analysis. From results found it could be concluded that this method outperforms a previous HPLC-MS based approach for the assessment of oxidative damage to proteins and DNA in urine as it fulfills specific requirements related to sample collection in preterm infants, and it combines an easy and quick sample preparation for the simultaneous determination of a set of metabolites capable to provide a snapshot of the oxidative status of the newborn.

The analytical approach, covering from sample collection to sample analysis, was successfully evaluated in a clinical study involving a total of over 200 urine samples from extremely low birth-weight infants previously described (see Introduction) [EUDRACT 2088-005047-42]. The high number of samples allowed to establish a set of reference ranges for o-Tyr, m-Tyr, p-Tyr, 3NO_2_-Tyr, 3Cl-Tyr, Phe, 2dG and 8OHdG concentrations in ELBW infants. In addition, we have also applied our method to healthy newborn patients to test its suitability and to establish the basal levels of the selected biomarkers in a control group. Interestingly, we were able to detect and quantify Basal levels of Phe, p-Tyr, o-Tyr, 3NO_2_-Tyr, 3Cl-Tyr and 8OHdG were determined. In contrast, basal levels of m-Tyr were below the LOD. Nonetheless, minimal values for m-Tyr in the urine samples of the preterm babies were respectively 5 times higher than the LOQs and 10 to 15 times higher than the LODs indicating that the sensitivity of the method was appropriate. The analysis of a larger cohort of healthy term babies is necessary to increase the robustness of the reference basal intervals, and will help to evaluate the need for higher preconcentration factors for the quantification of m-Tyr at basal levels. Further research will be carried out to assess the potential use of the panel of metabolites to establish an association between the type of treatment and the level of oxidative stress in the newborn. We hope that current efforts on the field will serve to define evidence-based recommendations for establishing optimal oxygen use during the resuscitation of preterm infants.

Whereas the method was specifically developed for the analysis of biomarkers of oxidative stress in urine samples from newborns it could be easily adapted to study responses related to oxidative stress in other fields such as the study of the effect of radio- and/or chemotherapy in cancer patients, or the evaluation of changes in the overall oxidative status upon nutritional interventions. Therefore, this method would have an impact on ongoing research aiming at a better understanding of the pathogenic mechanisms of disease or at improving predictions of the onset and disease progressions in newborns and especially preterm infants [37].

## Conclusions

This paper reports the validation of a novel, fast and straightforward UPLC-MS/MS method for the simultaneous quantification of a set of oxidative stress biomarkers formed by six protein and DNA oxidation products and three precursors, including o-Tyr, m-Tyr, p-Tyr, 3NO_2_-Tyr, 3Cl-Tyr, Phe, 2dG and 8OHdG in urine samples from newborns. Urinary concentration of these biomarkers provides highly reliable information on the oxidative status of these patients in the 12–24 hours previous to urine collection given the low glomerular filtration rate of preterm infants [38]. The method involves a single pretreatment step thus facilitating its application in clinical studies. In addition, the reduced sample volume required has rendered this method extraordinarily suitable for the assessment of the oxidation status in the highly vulnerable premature infants. This method could contribute to generate consistent results among different laboratories, which may in turn facilitate gaining insight into the effect of oxidative stress in both preterm and term infants.
